# Mitigation of DSS-Induced Colitis Potentially via Th1/Th2 Cytokine and Immunological Function Balance Induced by Phenolic-Enriched Buckwheat (*Fagopyrum esculentum* Moench) Bee Pollen Extract

**DOI:** 10.3390/foods11091293

**Published:** 2022-04-29

**Authors:** Sinan Chen, Yifei Xu, Ni Cheng, Feng Li, Haoan Zhao, Naisheng Bai, Hesham R. El-Seedi, Wei Cao

**Affiliations:** 1 College of Food Science and Technology, Northwest University, Xi’an 710069, China; chensinan111@163.com (S.C.); sophia1124125162@163.com (Y.X.); haoan_zhao@126.com (H.Z.); nsbai@nwu.edu.cn (N.B.); caowei@nwu.edu.cn (W.C.); 2 Bee Product Research Center of Shaanxi Province, Xi’an 710065, China; 3 School of Chemical Engineering, Northwest University, Xi’an 710069, China; lifeng@stumail.nwu.edu.cn; 4 Pharmacognosy Group, Department of Pharmaceutical Biosciences, Biomedical Centre, Uppsala University, P.O. Box 591, SE-751 24 Uppsala, Sweden; hesham.el-seedi@fkog.uu.se; 5 International Research Center for Food Nutrition and Safety, Jiangsu University, Zhenjiang 212013, China

**Keywords:** colitis, immunity, Th1/Th2, buckwheat bee pollen, phenolic compounds

## Abstract

Colitis is an inflammatory disease that results from the overactivation of effector immune cells, producing a high quantity of pro-inflammatory cytokines. Our study aimed to explore whether buckwheat (*F. esculentum*) bee pollen extract (FBPE) could inhibit the progression of dextran sulfate sodium (DSS)-induced colitis via regulating immune function. We isolated and identified six main phenolic compounds of FBPE such as luteolin (9.46 mg/g) by column chromatography, HPLC-DAD, ESI-MS and NMR spectroscopy, then assessed their effects on colonic mucosal injury by clinical symptoms, histomorphology and immunohistochemistry examinations. The results showed that FBPE at 25.2 g/kg body weight (g/kg BW) changed the clinical symptoms of colitis, the ICAM-1 expression in colon, the activity of related inflammatory mediators in colon tissue and helped restore the immune system. Compared with the model group (40.28%), the CD4 positivity was significantly reduced in the HD (High-dose group: 25.2 g FBPE/kg BW/day) group (20.45%). Administration of 25.2 g/kg BW of FBPE decreased the IFN-γ, TNF-α and IL-4 levels, while enhancing the IL-10 level, and significantly inhibited the abnormally decreased IgG (Model: 13.25 mg/mL, HD: 14.06 mg/mL), showing a reversal effect on the Th1/Th2 levels in colitis. These findings suggested that FBPE at 25.2 g/kg BW had the effects of alleviating colitis and immunomodulation, which can help in the development of safe and effective immune therapy.

## 1. Introduction

Colitis, a recurrent chronic inflammatory condition of the gastrointestinal system with disordered immune responses, is becoming more common around the world [1]. It is believed to be a multifactorial disorder involving oxidative stress, bacterial translocation through the intestinal mucosal barrier and dysregulation of immune responses, with an imbalance of pro-inflammatory (Th1) and anti-inflammatory (Th2) cytokines [2]. The development of colitis has been linked to a number of factors. Oxidative stress and colitis are clearly connected to the immunological response. Reactive oxygen species (ROS) levels promote cellular injury by affecting the activation and proliferation of T cells and the release of inflammatory cytokines, as macrophages, neutrophils, leading to an amplified mucosal inflammatory response and accelerated progression of the colitis [3,4]. CD4^+^ T cells differentiate into Th0 cells after antigen stimulation, then into helper T (Th) cell subsets (Th1, Th2, Th3, and Th17 cells), mediating the overproduction of some pro-inflammatory cytokines, for example, interferon (IFN)-γ and interleukin (IL)-1β in the colonic mucosa [5].

In view of the characteristics and pathogenesis of colitis, people are paying more attention to the use of safe and effective immune therapy, in addition to a healthy diet. For example, the addition of cheese to a diet [6], and dietary intake of the whole strawberry can suppress colitis via restoring immune homeostasis [7]. We previously studied the alleviative effect of rape bee pollen on DSS-induced colitis [8]. According to Song et al., caveolin-1 prevented dextran sulfate sodium (DSS)-induced colitis in mice by preventing mucosal barrier damage [9]. Lama et al. also applied DSS to study the immune-modulatory effects of oleoylethanolamide on colitis [10]. In addition, *Lactobacillus fermentum* species, *Inonotus obliquus* polysaccharide and *Bifidobacterium breve* inhibited the Th1 cell responses and regulated Th1/Th2 balance prior to the amelioration of DSS-induced colitis [11,12,13]. Moreover, many studies have demonstrated the inhibitory effect of dietary polyphenols on colitis [14,15]. Therefore, the regulatory mechanism of various interventions on colitis remains to be discussed, particularly since buckwheat bee pollen has been included in the list of new food resources in China [16], so it is more necessary to study it.

Nonetheless, to investigate whether buckwheat (*F. esculentum*) bee pollen extract (FBPE) could regulate immunity and exert protective effects on colitis, this paper analyzed the phenolic compounds of FBPE and examined its effects on colitis clinical symptoms, histological damage and immune-related parameters in mice, showing that FBPE not only suppressed colitis, but also reversed the imbalance of Th1/Th2 and protected intestinal mucosa, which is beneficial for protecting against colitis and developing safe and effective immune therapy.

## 2. Materials and Methods

### 2.1. Preparative and Phenolic Composition Analyses of FBPE

To prepare the FBPE, 2 kg of *Fagopyrum esculentum* Moench (*F. esculentum*) bee pollen collected directly from a local beehive in Yulin, Shaanxi Province, China (Supplementary Material Figure S1A) was percolated with 90% (*v*/*v*) ethanol (1:8) at room temperature for 2 days with constant stirring, then centrifuged (10 min, 4000 rpm), filtered, concentrated (rotary evaporation at 40 °C), and the extraction rate of FBPE was calculated to be up to 59.6%, and then stored at 4 °C until further analysis (Supplementary Material Figure S1B).

The phenolic composition is the main active compound in bee pollen, it is recognized for reducing oxidative stress, anti-inflammatory effects and immune enhancement. Determination of the total phenolic content (TPC) and total flavonoid content (TFC) was performed using the methods described in our previous study [17]. Additionally, the phenolic components of FBPE were analysed by high performance liquid chromatography (HPLC) with specific parameters, and the gradient elution procedures followed the same protocol applied in our previous study, where the separation and identification of these components had been done using thin layer chromatography (TLC) and chromatography, followed by pre-concentration and complete characterization (ESI-MS, NMR) [18].

### 2.2. Animals and Experimental Design

**Figure 1:**
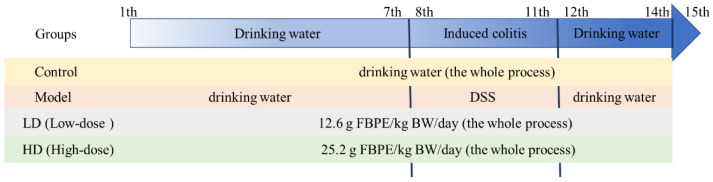
The experimental grouping and design.

All animal experiments were ethically approved and complied with the requirements of the Animal Ethics Committee of Northwest University. Forty-eight 18–22 g C57BL/6 male mice provided by Xi’an Jiaotong University School of Medicine (laboratory animal production license number: SCXK (Shaan) 2018-001; license: SYX (Shaan) 2015-002) were housed in a room with controlled temperature (22–25 °C) and humidity (50 ± 5%), and provided with alternating day and night light. The mice were acclimatized and housed for 1 week before the experiment. The experimental grouping and design are shown in Figure 1, where the doses were chosen based on our earlier investigations, and the induction of colitis was achieved by mice freely ingesting drinking water containing 3% *w/v* dextran sodium sulfate (DSS; 40,000 MW; Sigma, St. Louis, MO, USA). Additionally, mice in LD (Low-dose group: 12.6 g FBPE/kg BW/day) and HD (High-dose group: 25.2 g FBPE/kg BW/day) were given the corresponding dose of FBPE by gavage. Body weight, food intake, fecal consistency and fecal blood content of all animals were recorded daily throughout the trial period. The disease activity index (DAI), whose formula and scoring criteria were used to assess the severity of colitis, was used in accordance with the published reports [19]. On the 15th day, all mice were immediately sacrificed by cervical dislocation. Blood, colon and spleen were collected, then weights and lengths of colon and spleen were measured. Portions of colon were fixed in 10% formalin for histomorphometry and immunohistochemistry determination. A piece of the spleen was taken for the detection of the positive rate of immune cells. The remaining tissue samples were stored at −80 °C.

### 2.3. Histomorphometry and Immunohistochemistry (IHC)

Sections (1 cm) of colon were routinely dissected. According to the method described by Kumar et al. [20], and after modification, tissue samples were dehydrated in alcohol, embedded in paraffin, sectioned (4–5 μm), rehydrated and stained with hematoxylin and eosin (H&E). Finally, the effect of FBPE on colonic villi, glandular structure and crypt depth of colonic tissues were assessed by imaging with light microscopy (BK-FL, Optec Instrument Co., Ltd., Chongqing, China).

IHC of intercellular adhesion molecule-1 (ICAM-1) was performed to assess ICAM-1 expression in colon tissue according to the protocol described by Hamamoto et al. [21]. Briefly, after dewaxing, rehydration and antigen retrieval, tissue sections were incubated with a primary antibody (mouse anti-ICAM-1) and anti-mouse secondary antibody (1:200 dilution) for 60 min and 30 min, respectively, then counterstained with Mayer’s haematoxylin. Eventually, positive cells showed brownish-yellow cytoplasm and were counted by an investigator whose assignment to the group was unknown. Semi-quantitative scoring of colonic histological damage was performed according to the degree of tissue damage and cellular infiltration. Additionally, IHC score was calculated using the formula in the references [22].

### 2.4. Measurement of SOD, GSH-Px, MPO Activity and NO Level in Colon

Intercepted 0.1 g of the excised colons stored at −80 °C (*n* = 8 for each group) were homogenized in normal saline or corresponding reagents using a homogenizer (D-160, China) and their protein contents were quantified by a Protein Determination Kit (BAC-100, Jiancheng Biotech, Nanjing, China) for the following assays.

The biochemical kits purchased from Nanjing Jiancheng Biochemistry Co. (Nanjing, China) were used for the measurement of the superoxide dismutase (SOD), glutathione peroxidase (GSH-Px) myeloperoxidase (MPO) activity and nitric oxide (NO) level in the supernatant fraction, respectively, and the related specific procedures followed the manufacturer’s instructions. Among them, SOD activity was measured based on O^2−^ anions production, and this xanthine/xanthine oxidase method reported results with a value expressed as U/mg protein [23]. The degradation rate of the reduced form of nicotinamide adenine dinucleotide phosphate (NADPH) was used to assess the activity of GSH-Px, then the results were expressed as U/mg protein [24]. The O-dianisidine method was improved to measure MPO activity and to express the results as U/g colon weight [25]. According to the content of nitrite (NO^2−^) and nitrate (NO^3−^), the stable metabolites of NO, NO levels in colon tissue were measured and the results were shown as μmol/g protein [26].

### 2.5. Determination of GSH, GSSG and the Levels of T Cells in Splenocytes

The spleen was mashed into a single cell suspension and 0.4% trypan blue was added for counting. The collected splenocytes were washed twice with PBS buffer in the kit, centrifuged (1500 rpm/min) for 5 min at 4 °C, then suspended with 0.5 mL PBS buffer, and broken by ultrasound. The ratio of glutathione (GSH)-oxidized glutathione (GSSG) in the splenocytes was detected by a GSH detection kit (Nanjing Jiancheng Biochemistry Co., Nanjing, China), prior to the calculation of GSH/GSSG ratio.

Flow cytometry was used to examine Treg cells, following the method of Ami Ben Ya’acov et al. [27]. The supernatant was taken by centrifugation of the cell suspension, the pellets were resuspended in erythrocyte lysis buffer, and then washed with Hank’s solution to obtain splenocytes. The final concentration was adjusted to 2 × 10^5^/50 μL. Further, CD3-FITC, CD4-PE and CD8-PE-CY5 cell surface anti-mouse antibodies were used at saturating concentrations. A total of 1 × 10^6^ splenocytes were used in 100 mL PBS with 0.1% BSA for flow cytometry. The cells were then stained, completely washed (flow staining buffer), and finally analyzed with a BD FACS Canto II flow cytometer (Franklin Lakes, NJ, USA). FlowJo software was used to examine the data.

### 2.6. Determination of Immunoglobulin Content in Serum

Serum levels of immunoglobulins (IgG, IgA and IgM) were assessed based on a commercially available ELISA kit (Fusheng Testing Co., Ltd., Shanghai, China) using a double-antibody sandwich assay, as directed by the manufacturer. Briefly, 1 mL whole blood samples were naturally coagulated for 10–20 min, and centrifuged (2500 rpm, 20 min), then the supernatant was used to measure the three kinds of immunoglobulin, with the results shown as mg/mL.

### 2.7. Quantitative Real-Time PCR (qPCR) Analysis

The TaKaRa Mini-BEST kit (TaKaRa Bio INC., Shiga, Japan) and PrimeSciptTM RT Master Mix kit (TaKaRa Bio INC., Japan) were used for the RNA extraction and cDNA synthesis, respectively. The sequences of the gene expression primers required for amplification are summarized in Table 1. Finally, amplification of DNA was completed by TB Green chimeric fluorescence using a real-time PCR instrument (Gentier 96E, Xi’an Tianlong Science and Technology Co., China), and the relative mRNA expression of genes (TNF-α, IFN-γ, IL-4 and IL-10) was calculated by the 2^−ΔΔCt^ method [28].

### 2.8. Statistical Analysis

All tests were repeated three times and the resulting values were expressed as mean ± SD. Data were analyzed using a SAS one-way ANOVA (version 8.1, SAS Institute, Cary, NC, USA), then Duncan’s multiple range test was performed. A *p* value < 0.05 was considered statistically significant.

## 3. Results and Discussion

### 3.1. Phenolics Composition of FBPE

Buckwheat bee pollen is now commercially consumed as a dietary supplement precisely because of its outstanding nutrition, especially the highly regarded phenolics. Table S1 summarized the results obtained for the phenolic composition, with TPC reaching 18.59 mg gallic acid/g, and TFC showing 16.35 mg rutin/g. Then we identified and quantified the phenolic compounds of buckwheat bee pollen, we isolated six main phenolic compounds by column chromatography, and they were characterized as luteolin, resveratrol, kaempferol, caffeic acid, chlorogenic acid andcatechin, as detected by HPLC-DAD, ESI-MS and NMR spectroscopy [18], and the detailed information is shown in the Supplementary. The compounds 1–6 have a total of 25.7 mg/g weight. Among them, luteolin reached 9.46 mg/g, which was the highest content of the quantified phenolic compounds. Resveratrol followed with 5.25 mg/g, while kaempferol and caffeic acid were more than 3 mg/g (Supplementary Material Figure S2 and Table S1). Moreover, the structure diagrams of the six identified main phenolic compounds are shown in the supplementary (Supplementary Material Figure S3). These showed that buckwheat bee pollen contained a higher content of phenolics, and the TPC was higher than that of many studied bee pollens such as the Moroccan plant origin of *Spiraea salicifolia* (8.07 mg gallic acid/g) [29], palm bee pollen from Sonoran Desert (15.91 mg gallic acid/g) [30], Romania harvested from several floral origins (e.g., *Helianthus annuus* L. was 11.4 mg gallic acid/g, *Pinus* sp. was 6.4 mg gallic acid/g) [31], and also higher than that of buckwheat bee pollen collected from Konya (7.12 mg gallic acid/g) [32]. This may be due to the plant characteristics of buckwheat itself having a rich synthesis of phenolics, its early documented medicinal value also proves this [33]. Even if the pollen source plant is the same buckwheat, the phenolic composition varies due to environmental influences such as geographic regions and the time of collection. It can be seen that buckwheat bee pollen rich in six phenolic compounds, especially luteolin, has certain research value in the field of antioxidation and inflammation. In addition, luteolin is an essential flavone widely found in several plants, and its beneficial functions have been confirmed by a number of studies [34]. A study by Lee et al. indicated that luteolin found in Korean *Papaver* *rhoeas* bee pollen was the most effective inhibitor of neuraminidase [35], and a study on the phenolic profile of ethanol extracts of sunflower pollen from Serbia (TPC was 244.4 mg/kg) showed the antioxidant properties of luteolin (1.22 mg/kg) [36]. Different types of phenolics can target binding to different receptors on immune cells, which undergo changes that stimulate intracellular signaling pathways to modulate the host immune response. For example, resveratrol has been shown to exert immunomodulatory effects by directly targeting central cellular immune components such as dendritic cells, reducing the expression of activating receptors CD28 and CD80 on immune cells and increasing the production of the immunosuppressive cytokine IL-10. Meanwhile, resveratrol has also been found to exert anti-tumor and immune-enhancing effects by targeting Sp1 as a molecular target. The receptors of certain flavonoids on T cells are Toll-like receptors, whose connections can activate nuclear factor-(NF-) κB, Akt and other pathways, which in turn activate dendritic cells. Regulatory T cells (Tregs) contribute to the maintenance of immune tolerance [37].

### 3.2. FBPE Attenuated the Clinical Symptoms of DSS-Induced Colitis

**Figure 2:**
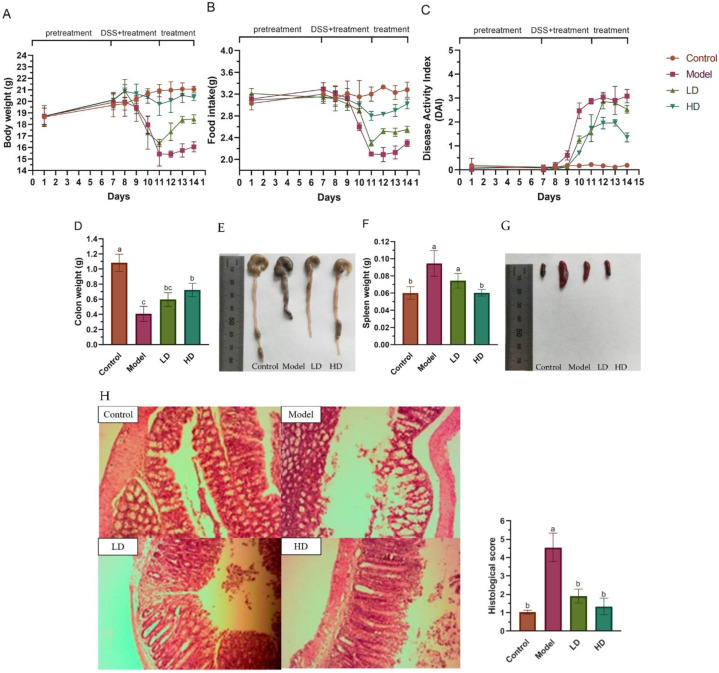

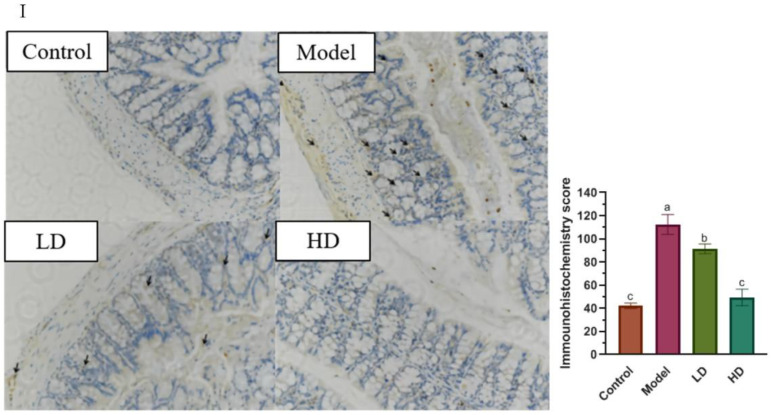
Effects of FBPE on colitis symptoms and colonic lesions, including body weight (**A**), food intake (**B**), disease activity index (DAI) (**C**), colon weights (**D**), typical images for length comparison of colon samples (**E**), spleen weights (**F**), typical images for swelling level comparison of spleen samples (**G**), H&E staining of colon sections and its semi-quantitative histological scoring (**H**), ICAM-1 immunohistochemical colon sections and score (**I**). Arrows represent the expression of ICAM-1. Different lower-case letters correspond to a significant difference of *p* < 0.05.

Before the onset of DSS-induced colitis (days 1–7 of the experiment), there were no significant differences in body weight, food intake and DAI between the four groups. During the DSS induction period (days 8–11), colitis model mice lost significant body weight compared to the control group, while high-dose FBPE administration alleviated the DSS-induced weight loss (Figure 2A). The food intake in the model group of colitis mice was markedly decreased from the 9th day. Although similar in the LD group, it was mild, and the symptoms were effectively alleviated in the HD group (Figure 2B). The significant reduction of DAI in the groups treated with different doses of FBPE compared with the model group reflected the alleviating effect of FBPE on body weight, blood in stool and fecal consistency in colitis mice (*p* < 0.05) (Figure 2C). During FBPE treatment after DSS induction (12–14th days), body weight, food intake and DAI were effectively alleviated in both FBPE treatment groups and showed a dose-effect correlation.

Meanwhile, colitis is also accompanied by shortening of the colon [38]. Compared with the controls, the colon weight of colitis mice was significantly decreased (Figure 2D), and the colon length was also noticeably shortened (Figure 2E) (*p* < 0.05). When colitis strikes, the spleen, an important immunological organ, swells [39,40], and the consumption of FBPE reduced the degree of spleen swelling (Figure 2G). While the colon is the site of disease initiation, the regulation of the inflammatory cytokine release can be mediated via the spleen, and when the body is stimulated by antigen, the activation and proliferation of lymphocytes in the spleen lead to the proliferation of lymphoid tissue and the weight gain of spleen [41]. Treatment of colitis mice with two different doses of FBPE reversed the DSS-induced increase in spleen weight (*p* < 0.05), especially the weight of colon and spleen in the HD group was markedly different from that in the model group, showing noticeable recovery (Figure 2D–G). Thus, the FBPE diets of 12.6 g/kg BW and 25.2 g/kg BW can ameliorate these pathological symptoms of DSS-induced colitis, with the latter having a greater impact.

Colon histological evaluation showed that DSS injury caused colon specimens of the model group to exhibit scattered erosions, in detail, the epithelial barrier, villi and glandular structures all exhibited abnormalities. In LD group, fewer erosions were observed, although occasional erosions still existed in the colon, while the colonic histological structure of the HD group had largely recovered to the level of the control mice. Histopathological scores were also significantly reduced in the two FBPE treatment groups to the point that they were not dramatically different to controls (Figure 2H). Phenolics have been studied to improve abnormal crypt lesions by reducing abnormal crypts [42].

ICAM-1 expression was studied by immunostaining in the four groups (Figure 2I). DSS toxicity caused ICAM-1 to be strongly expressed in the mucosal and submucosal vascular endothelium, whereas in the colonic epithelium of control mice there were only few ICAM-1-positive cells, and the LD group had a slight expression. More strikingly, compared to model group, mice treated with 25.2 g/kg BW of FBPE had a noticeably lower percentage of ICAM-1 positive cells in the colonic epithelium, and the immunohistochemical score of HD group was largely consistent with the controls (*p* > 0.05). The occurrence of colitis has been proven in recent research to be possibly associated with an unbalanced immune response, including to luminal or epithelial antigens or to other external factors. Some studies have proved that polyphenols can inhibit the expression of ICAM-1, which is essential for the influx of neutrophils into the colonic mucosa, thus influence the immune response to relieve colitis [43]. For example, Narisara et al. found that the nutlets of Thai *perilla* had the effect of decreasing the expression of ICAM-1 precisely owing to their phenolic acids and flavonoids [44]. Polyphenol-rich grape pomace extracts also had the same effect [45]. Therefore, the present results demonstrated the potential utility of polyphenol-rich FBPE in the treatment of colitis, which may also be related to other bioactive compounds in FBPE and their possible synergistic effect with phenolics.

### 3.3. Effect of FBPE on Inflammatory Mediators in Colon

**Figure 3:**
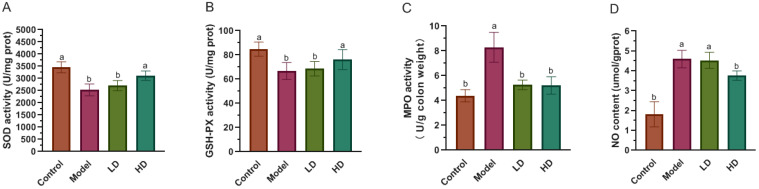
Effect of FBPE on the inflammatory mediators in the mice colon with DSS-induced colitis. SOD activity (**A**), GSH-Px activity (**B**), MPO activity (**C**) and NO content (**D**). Different lower-case letters correspond to a significant difference at *p* < 0.05.

The present results revealed that the experimental group intervened by FBPE produced statistically significant differences in colon tissue enzymatic (SOD, GSH-Px and MPO) antioxidant activity and NO content in comparison to the model mice (Figure 3). Although ROS represent a defense mechanism, damage to the immune system of the organism occurs when there is an excess of ROS, reflected by a decline of SOD and GSH-Px activity [46,47], which is due to the decrease in SOD activity with the onset of inflammatory diseases and the production of other forms of carbon, nitrogen and oxygen-centered radicals from excess superoxide anions. In addition, SOD presence may suggest cell membrane malfunction and an anti-inflammatory response [48,49]. In the present study, SOD activity was significantly reduced in model group (*p* < 0.05), while the SOD activity of HD group was 3113.6 U/mg prot, which was 1.23-fold of that in the model group. FBPE at 25.2 g/kg BW mitigated the decreased of SOD activity (*p* < 0.05) (Figure 3A). Similarly, GSH-Px is an enzyme that prevents lipid peroxidation in tissues. GSH-Px catalyzes the transformation of H_2_O_2_ to harmless byproducts to prevent cellular destruction. During H_2_O_2_ scavenging, GSH is oxidized to GSSG by the enzyme GSH-Px [50]. Figure 3B showed that FBPE administration improved the significant reduction of GSH-Px activity. An 14.2% increase of GSH-Px activity in the colon of HD mice was observed in comparison to the model group (*p* < 0.05). According to a growing number of studies, GSH-Px is closely associated with the body’s immunity due to its involvement in T-cell development. Kim et al. claimed that enhancement of regulatory T-cell function can effectively reduce DSS-induced colitis [51]. Moreover, recent studies have reported that polyphenols could enhance immune enzyme activity in experimental animals [52,53]. They showed that tissue damage and impaired antioxidant immune system during colonic inflammation led to a decrease in SOD and GSH-Px activity. Therefore, FBPE at 25.2 g/kg BW could mitigate the reduction of SOD and GSH-Px activities, and assist in restoring the immune system and relieving colitis.

Conversely, MPO reflects the degree of neutrophil infiltration, marks the functionality of neutrophils and is directly related to inflammation [54]. As shown in Figure 3C, colonic MPO activity was increased by 47.1% in the model mice. FBPE administration in different dosages could significantly reduce the activity of MPO to control level (*p* < 0.05). Moreover, NO has a role in mediating the pathological effects of cytokines like TNF-α, IL-6 and IL-1β, and is therefore seen as a new immune molecule and inflammation mediator [55]. However, when it is produced in excess, it can cause colitis. It is well known that a large amount of NO is produced by iNOS, and the occurrence of inflammation and tissue damage may be caused by the NO and the activation of oxygen free radicals [56,57]. The result is shown in Figure 3D, the NO content was significantly elevated in the model group, which was 2.5 times higher when compared to the controls. Despite the fact that there was no remarkable difference between the LD and model groups, the HD group was able to considerably lower NO level in colitis mice. (*p* < 0.05), indicating that FBPE at 25.2 g/kg BW could regulate the immune system by inhibiting NO production and thus reduce colitis.

### 3.4. Effect of FBPE on Immune Response in Splenocytes and Colon

**Figure 4:**
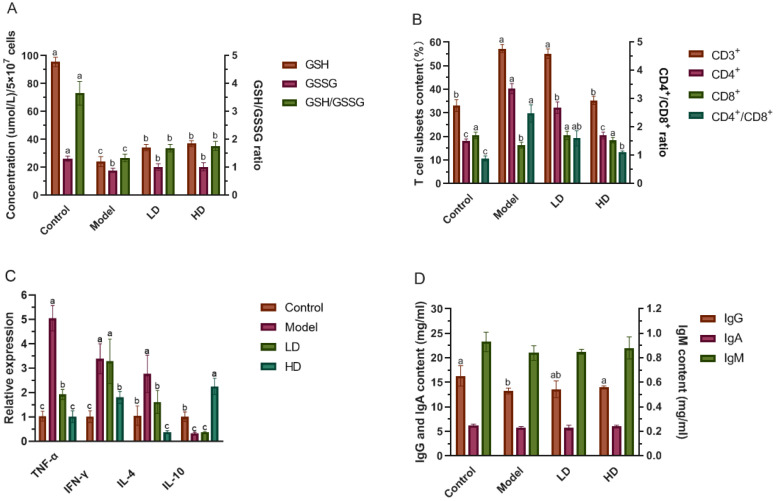
Effect of FBPE on immune response in splenocytes, colon and serum of DSS-induced colitis. GSH, GSSG content and GSH/GSSG ratio in splenocytes (**A**), T cell subsets content in splenocytes (**B**), inflammatory cytokines mRNA levels in colon (**C**), immunoglobulin (IgG, IgA and IgM) content in serum (**D**). Different lower-case letters correspond to a significant difference at *p* < 0.05.

The sustained production of ROS in colitis imbalances oxidative stress, which in turn accelerates the progression of inflammatory disease, involving an immune response [58] and exhibits excessive depletion and deficiency of antioxidants such as GSH [59]. GSH is the main redox buffer in most cell types and its reduced form (GSH) constitutes the first line of cellular defense against antioxidant damage in order to avoid the attack of important cellular components by ROS [58]. The final result of a balance between GSH synthesis and the combined rate of GSH consumption by ROS and excretion of the resultant GSSG is intracellular GSH concentration [60,61]. Therefore, we determined the amount of GSH, GSSG and the GSH/GSSG ratio in splenocytes to measure the redox status of the system, due to the fact that chronically low levels of GSH/GSSG ratio can be a valid indicator of oxidative stress [62]. The results showed that the LD and HD groups could restore the GSH content in splenocytes to 35.8% and 38.8% of the control group, respectively. The GSH/GSSG ratio in the model mice was only about one-third of that in the controls (control: 3.65, model: 1.34). Both 12.6 g/kg BW and 25.2 g/kg BW FBPE treatments reversed this decrease (LD: 1.70, HD: 1.77) (Figure 4A).

According to previous studies, three key intracellular redox systems, NADPH/nicotinamide-adenine dinucleotide phosphate (NADP), thioredoxinred/thioredoxinox and GSH/GSSG, maintained the production of efficient immune responses, with the highest cellular GSH levels, which were 1000 times higher than other redox couples, indicating the importance of the GSH level [63]. Shuji Kojima et al. showed that the balance of cellular GSH was regulated to improve immunological functions [64]. Recent developments in basic immunology have also revealed the importance of GSH and cellular redox balance in the generation of an immune response. Therefore, FBPE can effectively maintain the balance between intracellular redox couple (GSH/GSSG) and enhance immune function.

Next, we searched for possible differences in total T lymphocytes (CD3^+^) and their subsets (CD4^+^ and CD8^+^ T cells population) in splenocytes of colitis mice and the regulation of the immune system by FBPE. Inappropriate down regulation of an activated immune system is considered as the main pathogenetic mechanism of colitis. Different cell types induce intestinal pathology, including T cell dysregulation, which results in an imbalance between activated and regulatory T cells. Different cell types induce intestinal pathology, involving T cell dysregulation, which results in an imbalance between activated and regulatory T cells [65]. The spleen is an important immune organ, rich in a large number of lymphocytes, which serves as a regulator of CD4^+^ T cells and transmits antigenic information in the immune response. CD8^+^ T cells are inhibitory and killer T cells, which have the function of inhibiting CD4^+^ T cells, and immunosuppressive effects. As shown in Figure 4B, the CD3^+^ of the model group (57.30%) was found to be markedly higher than the CD3^+^ of the control group (33.15%), but 25.2 g/kg BW of FBPE treatment (35.35%) reversed this increase. Among them, flow cytometric examination revealed a significant drop in CD4^+^ of the FBPE administered mice compared to the model mice (LD: 32.25%, HD: 20.45%), although these proportions were still greater than those in the controls (18.15%). Amount and activity of regulatory CD4^+^ T cells affect the disease resistance of the organism [66]. In addition, as seen by flow cytometry data, splenocytes CD8^+^ was significantly elevated with FBPE administration compared to the model group (LD: 20.43%, HD: 18.45%), and not significantly different from the controls (20.48%). This was the same as the activation state of CD8^+^ in colitis-associated colon cancer studied by Olguín et al. [65]. Regulatory CD8^+^ T cells, which include functional subsets characterized by cytokine, metabolic, and cytotoxic mechanisms of action, assist in the reduction of colitis and other inflammatory disorders, although they have been less investigated [67,68]. It can be speculated that FBPE can regulate CD8^+^, regulate immunity and alleviate colitis to prevent the occurrence of colon cancer. The ratio of CD4^+^/CD8^+^ is an important index reflecting the activity of T lymphocytes. The HD group reduced this ratio to 1.11, which was significantly lower than the colitis mice, although there was still a difference among the HD and control group. Detailed data on flow cytometry analysis can be found in supplementary Figure S4. These results confirmed that the protective role of 25.2 g/kg BW of FBPE in colitis may be partly mediated by regulating T cell activity.

CD4^+^ T cells are responsible for orchestrating adaptive immune responses, and when activated by T cell receptors, they develop into distinct Th lineages including Th1, Th2, Th17, and regulatory T cells, based on the cytokine milieu in the microenvironment [69]. Th1 cells release cytokines that cause inflammation and antiviral effects, such as IL-2, TNF-α, and IFN-γ, that contribute to T-cell-mediated immune responses. Th2 cells can produce cytokines (IL-4, IL-10, IL-13 et al.) that promote humoral immune responses. To put it another way, stimulated immune cells produced inflammatory cytokines like IL-4 uncontrolled in DSS-induced colitis pathology [70], which activated inflamed immune cells and generated an immune imbalance, eventually leading to a prolonged inflammatory response in colon tissue [71]. The findings of this investigation revealed that DSS administration to mice increased the IFN-γ, TNF-α and IL-4 levels, while reducing the IL-10 level in colon tissue (Figure 4C). TNF-α expression in the LD and HD groups was significantly reduced by 61.9% and 79.8%, respectively, when compared to the model group. IFN-γ expression was remarkably decreased by 47% in the HD group, and IL-4 expression was dramatically downregulated in FBPE treated groups. DSS induction reduced the IL-10 expression in the colitis mice to 33% of the controls, while it was up-regulated to 6.8 times of the model group in the HD group. Many studies have confirmed the pathogenic effect of IFN-γ and TNF-α in colitis, where they are elevated at the site of IL-8 and monocyte chemoattractant protein (MCP)-1, as well as post-activation regulation, inducing normal T cell expression and secretion, which in turn promotes the progression of inflammatory infiltrates [72,73]. Furthermore, IL-10 is a kind of anti-inflammatory cytokine with intestinal immunomodulatory effects, and its level decreases with increasing mucosal infiltration of inflammatory cells and the degree of colitis [74]. A study on IL-10-deficient mice found a significant increase in intestinal permeability prior to the onset of colitis, and thus relief of colitis could be achieved by improving intestinal epithelial barrier function, suggesting that decreased expression of IL-10 alters intestinal permeability, thereby exacerbating the development of colitis [75], and FBPE could alleviate colitis by increasing the expression of IL-10, and lead to a decrease in mucosal barrier disruption as well as a decrease in TNF-α and ROS.

The findings of the present study were in accordance with most studies demonstrating that an excessive Th1 T cell response or an excessive Th2 T cell response caused colitis, both featuring increased IFN-γ/TNF-α production and decreased IL-4/IL-10 production, respectively, and 25.2 g/kg BW of FBPE can effectively resist DSS-induced colitis by affecting this classical Th1 and Th2 response.

### 3.5. Effect of FBPE on Immunoglobulin in Serum

Abnormal Th1 and Th2 responses are accompanied by changes in serum IgM, IgA and IgG. In assessing inflammation and immunity, we chose to analyze immunoglobulin levels in the mice serum using ELISA as a way to determine the anti-inflammatory effect of FBPE on immunoglobulin expression levels. Among them, IgG is the most important antibody component in serum and extracellular fluid, and also the most abundant immunoglobulin in serum. Figure 4D shows that the serum IgG content was dramatically decreased (*p* < 0.05) in mice due to DSS induction. The 25.2 g/kg BW of FBPE increased the serum IgG content (14.06 mg/mL) to control levels (16.34 mg/mL) (*p* > 0.05). But beyond that, no significant effect of either DSS or FBPE on IgA and IgM contents in the serum were observed (*p* > 0.05), compared with controls. Studies have shown that DSS exerts direct toxicity on the colonic mucosal epithelium, leading to epithelial cell and barrier function damage, indicated by high intestinal permeability, causing the IgG and IgA to participate and form local immune complexes in the mucosa [76]. Therefore, damaged epithelia in the inflamed colon enhanced leakage of IgG into the lumen, which could explain the lower serum levels of IgG in the colitis mice [77], while FBPE may improve intestinal permeability by controlling the Th1/Th2 balance and increase serum IgG level. In addition, its phenolics may enhance intestinal mucosal immunity by enhancing the number of intraepithelial T cells and mucosal eosinophils [78], and it has been shown in many studies that phenolics can alter the immune index of immunoglobulins [79]. The results of this study showed that 25.2 g/kg BW of FBPE significantly inhibited the abnormally decreased IgG, suggesting that the mechanism for the treatment of colitis is linked to the effective regulation of an abnormal immune response and intestinal permeability.

## 4. Conclusions

In summary, FBPE has a rich in nutritional composition, among which six main phenolic compounds have been isolated and identified, where the content of luteolin was as high as 9.46 mg/g, resveratrol was 5.25 mg/g and kaempferol was 3.67 mg/g. Furthermore, FBPE could ameliorate clinical symptoms and histological damage in colitis mice, which may be due to regulating immunological function, and reversing the imbalance of Th1/Th2, thereby exerting its protective effect on the colon. In particular, 25.2 g/kg BW of FBPE could enhance IL-10 expression in the colon and the IgG level in serum, improving epithelial barrier function and protecting the intestinal mucosa. These findings prompted us to further explore and reveal the effects of purified bee pollen phenolics on colitis and immune-related mechanisms. Nevertheless, our findings suggest that FBPE will be an excellent natural product candidate in combating chronic inflammatory disorders such as colitis.

## Figures and Tables

**Table 1:** Primer sequences for qPCR.

Primer	F	R
TNF-α	5′-AGCCGATGGGTTGTACCTTG-3′	5′-AGTACTTGGGCAGATTGACCTC-3′
IFN-γ	5′-AGGTCCAGCGCCAAGCATTCAA-3′	5′-AGCAGCGACTCCTTTTCCGCTT-3′
IL-4	5′-AACGTCCTCACAGCAACGAA-3′	5′-AGGCATCGAAAAGCCCGAAA-3′
IL-10	5′-CAGTACAGCCGGGAAGACAA-3′	5′-CCTGGGGCATCACTTCTACC-3′
β-actin	5′-CACGATGGAGGGGCCGGACTCATC-3′	5′-TAAAGACCTCTATGCCAACACAGT-3′

## Data Availability

The data presented in this study are available in Supplementary Material.

## Supplementary Materials

The following supporting information can be downloaded at: https://www.mdpi.com/article/10.3390/foods11091293/s1, Figure S1: Buckwheat (*F. esculentum*) bee pollen sample (A) and FBPE (B). Figure S2: High performance liquid chromatography (HPLC) chromatograms of phenolic standards (A) and FBPE (B) at 210 nm. Chlorogenic acid (1), Caffeic acid (2), Resveratrol (3), Catechin (4), Luteolin (5) and Kaempferol (6). Figure S3: Structural identification of the six main phenolic compounds in FBPE. Figure S4: Representative images showing flow cytometry of T cell subsets in different groups of splenocytes. Table S1: Phenolic compounds of FBPE.
