# Supplementary material for: Mitigation of DSS-Induced Colitis Potentially via Th1/Th2 Cytokine and Immunological Function Balance Induced by Phenolic-Enriched Buckwheat (Fagopyrum esculentum Moench) Bee Pollen Extract
Source: Foods. 2022 Apr 29;11(9):1293. doi: 10.3390/foods11091293 (PMC9105923; doi:10.3390/foods11091293)
Supplement: Supplementary file 1 [file foods-11-01293-s001.zip › foods-1666754-supplementary.pdf]

Supplementary

Figure captions

Figure S1 Buckwheat (*F. esculentum*) bee pollen sample (A) and buckwheat (*F. esculentum*) bee pollen extract (FBPE) (B).

Figure S2 High performance liquid chromatography (HPLC) chromatograms of phenolic standards (A) and FBPE (B) at 210nm. Chlorogenic acid (1), Caffeic acid (2), Resveratrol (3), Catechin (4), Luteolin (5), Kaempferol (6).

Figure S3 Structural identification of 6 main phenolic compounds in FBPE.

Figure S4 Representative images showing flow cytometry of T cell subsets in different groups of splenocytes.

A B

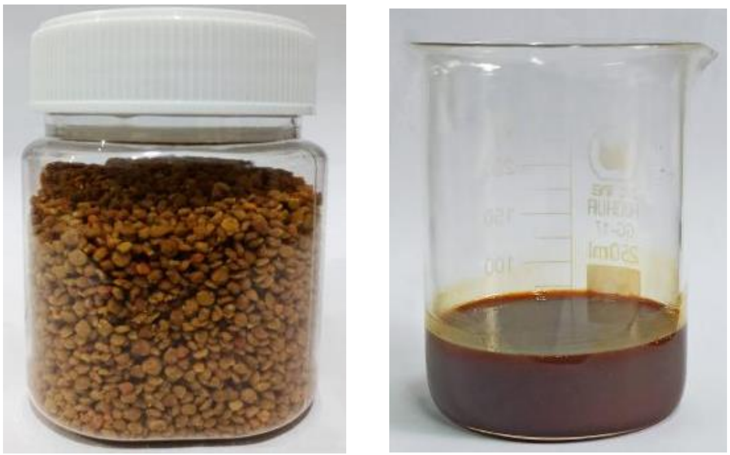

Figure S1. Buckwheat (*F. esculentum*) bee pollen sample (**A**) and buckwheat (*F. esculentum*) bee pollen extract (FBPE) (**B**).

A B

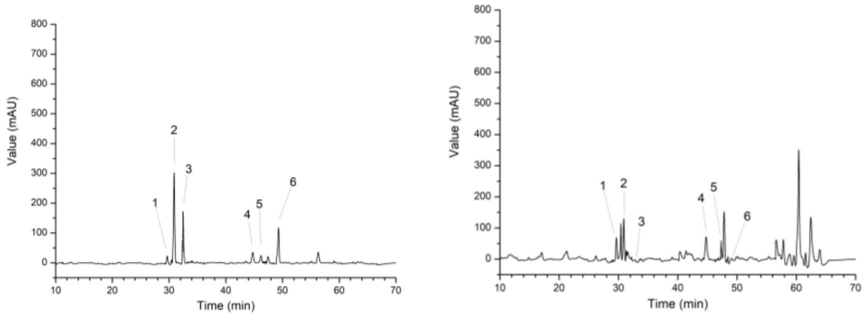

Figure S2. High performance liquid chromatography (HPLC) chromatograms of phenolic standards (**A**) and FBPE (**B**) at 210nm.

Chlorogenic acid (1): ^{1}H-NMR (400 MHz, methanol-d_4): δ 7.48 (1H, d, *J*= 15.9 Hz, H-7), 6.97 (1H, d, *J*= 2.0 Hz, H-6), 6.88 (1H, dd, *J*= 2.0, 8.1 Hz, H-2), 6.70 (1H, d, *J*= 8.2 Hz, H-5), 6.19 (1H, d, *J*= 15.9 Hz, H-8), 5.25 (1H, d, *J*= 4.2 Hz, H-5′), 4.08 (1H, s, H-1′), 3.65 (dd, *J*= 8.4, 3.1 Hz, H-6′), 3.30~3.20 (2H, m, H-2′), 2.03 (2H, d, *J*= 5.2 Hz, H-4′). ^{13}C-NMR (400 MHz, DMSO-d_6): δ 127.74 (C-1), 115.12 (C-2), 149.58 (C-3), 147.08 (C-4), 116.43 (C-5), 123.00 (C-6), 146.79 (C-7), 115.19 (C-8), 168.62 (C-9), 71.95 (C-1′), 38.17 (C-2′), 73.38 (C-3′), 38.69 (C-4′), 71.22 (C-5′), 76.09 (C-6′), 177.03 (C-7′).

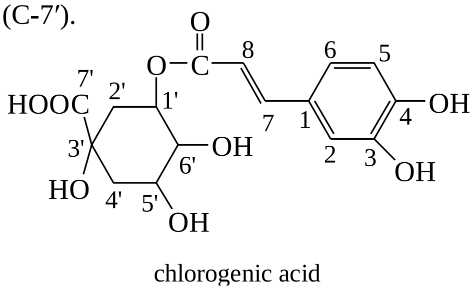

chlorogenic acid

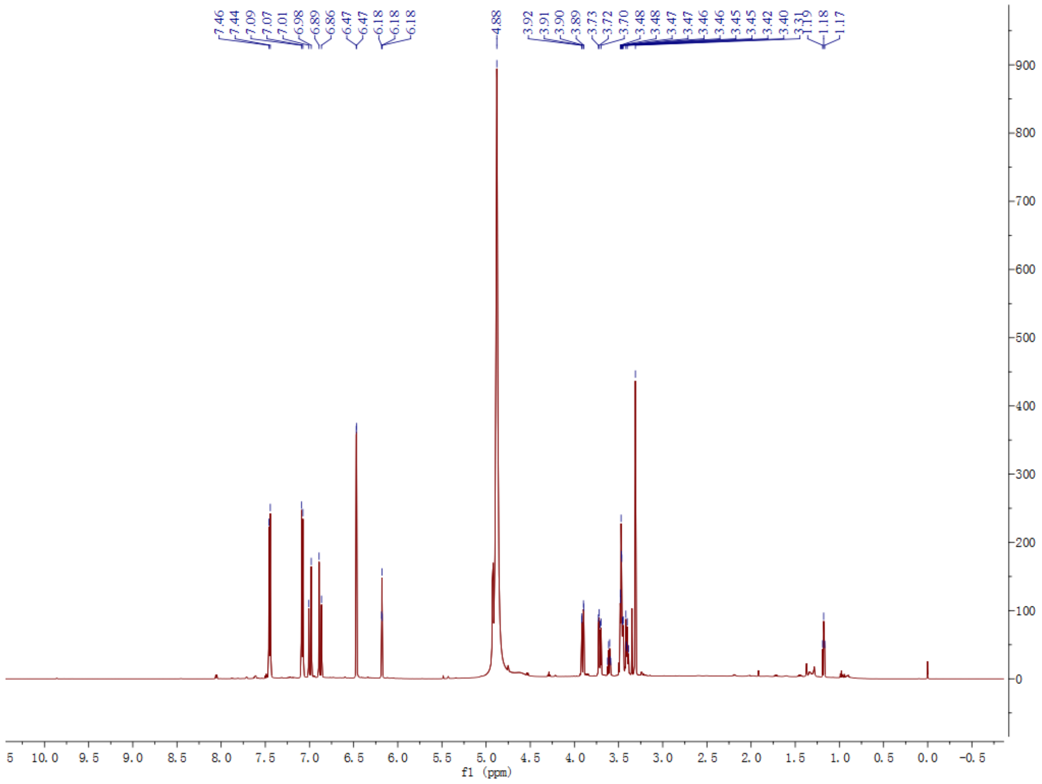

The ^{1}H-NMR spectrum of compound 1

Caffeic acid (2): ^{1}H-NMR (400 MHz, DMSO-d_6): δ 6.97 (1H, d, *J*= 2.0 Hz, H-3), 7.02 (1H, d, *J*= 2.0 Hz, H-5), 6.95 (1H, d, *J*= 2.0 Hz, H-6), 7.41 (1H, d, *J*= 15.9 Hz, H-7), 6.75 (1H, d, *J*= 8.1 Hz, H-8), 12.16 (1H, s, -COOH), 6.17 (1H, d, *J*= 15.9 Hz, -OH×2). ^{13}C-NMR (400 MHz, DMSO-d_6): δ 127.75 (C-1), 116.46 (C-2), 146.77 (C-3), 149.44 (C-4), 115.04 (C-5), 122.86 (C-6), 147.05 (C-7), 115.47 (C-8), 171.04 (C-9).

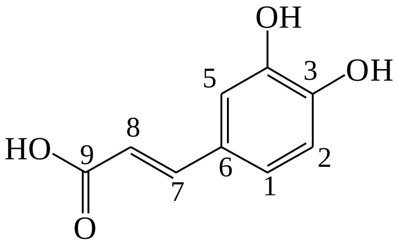

caffeic acid

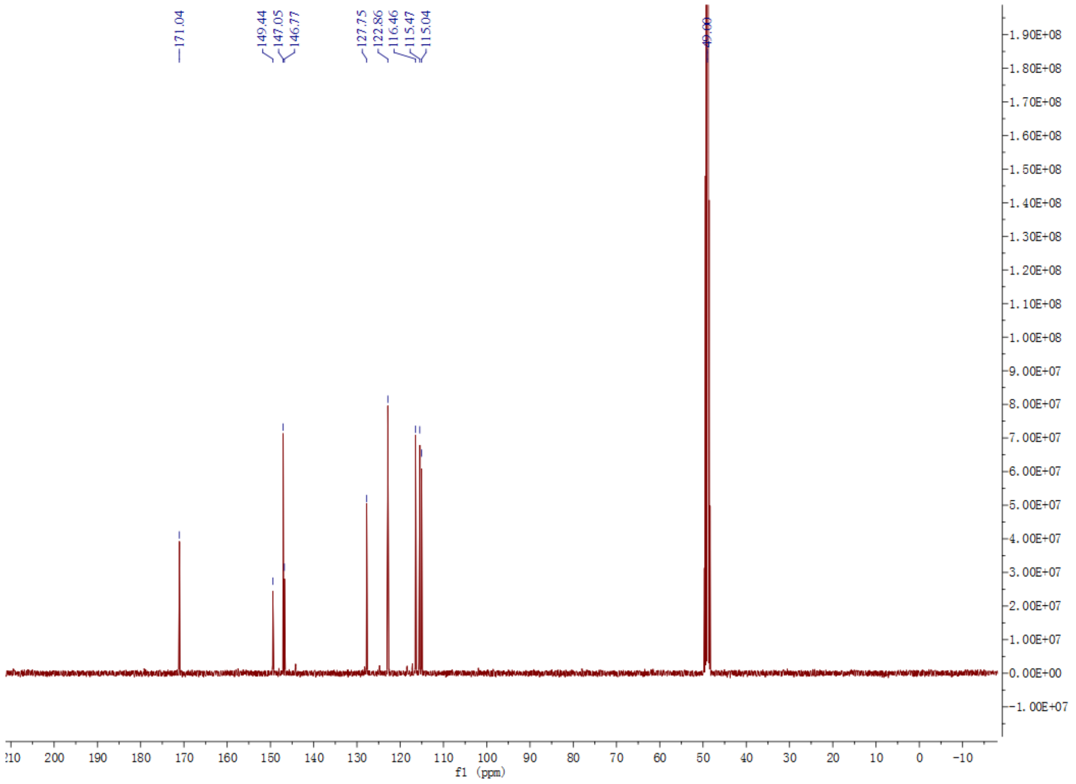

The ^{13}C-NMR spectrum of compound 2

Resveratrol (3): ^{1}H-NMR (400 MHz, DMSO-d_6), δ 6.38 (1H, d, 1.6 Hz, H-2&6), 6.11 (1H, t, 2.0 Hz, H-4), 7.34 (2H, d, 8.4 Hz, H-2′&6′), 6.75 (2H, d, 8.4 Hz, H-3′&5′), 6.93 (1H, d, 16.4 Hz, H-7′), 6.87 (1H, d, 16.4 Hz, H-8′), 9.24 (s, H-3&5-OH), 9.60 (1H, s, H-4′-OH). ^{13}C-NMR (400 MHz, DMSO-d_6): δ 139.4 (C-1), 104.4 (C-2&6), 158.6 (C-3&5), 101.8 (C-4), 128.1 (C-1′), 127.9 (C-2′&6′), 115.6 (C-3′&5′), 157.3 (C-4′), 127.9 (C-7′), 125.7 (C-8′).

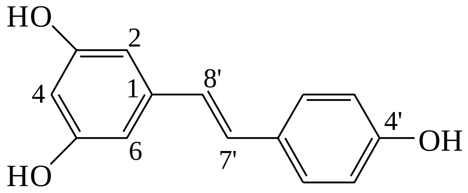

Resveratrol

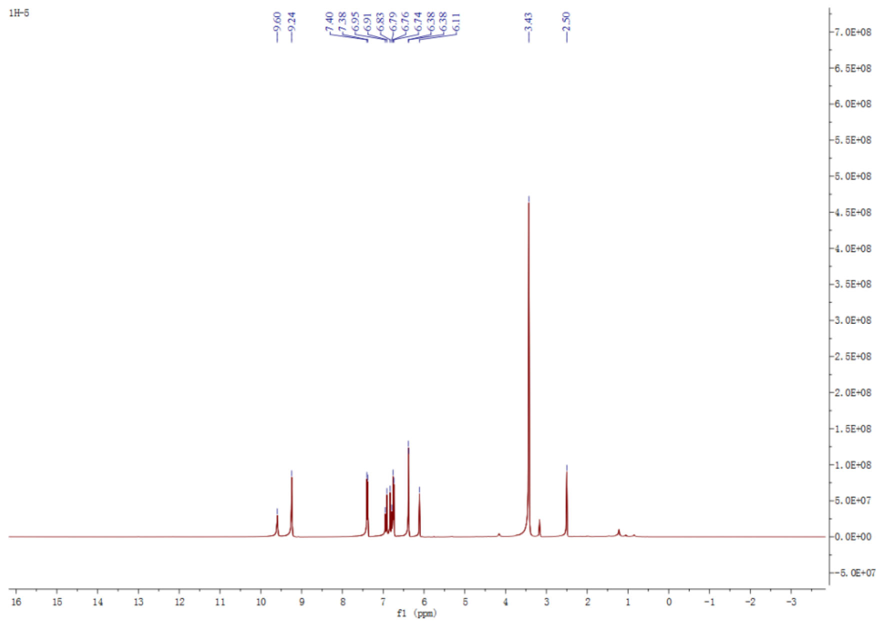

The ^{1}H-NMR spectrum of compound 3

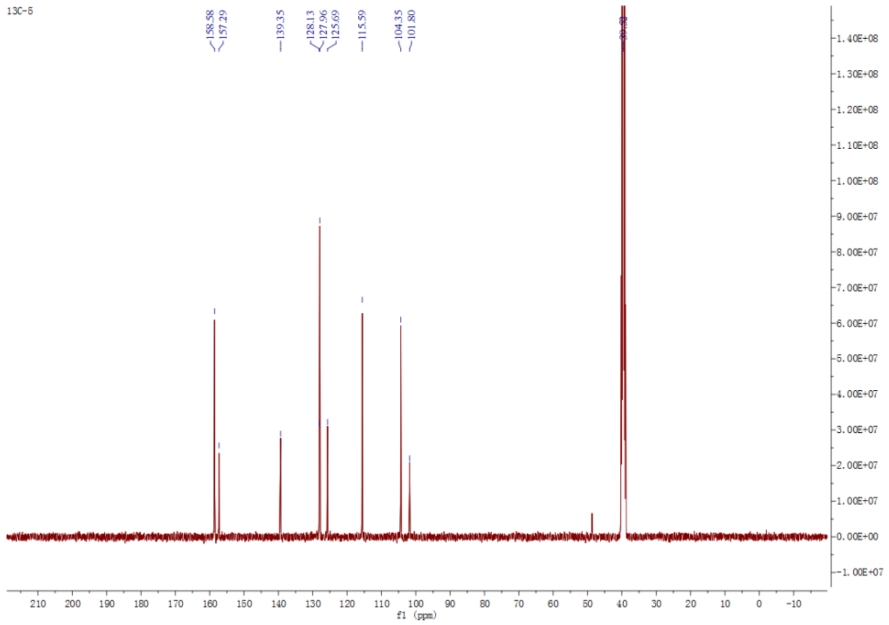

The ^{13}C -NMR spectrum of compound 3

catechin (4): ^{13}C-NMR (400 MHz, DMSO-d_6): δ 83.20 (C-2), 68.97 (C-3), 28.99 (C-4), 188.99 (C-5), 96.17 (C-6), 184.87 (C-7), 95.46 (C-8), 108.21 (C-9), 100.80 (C-10), 132.51 (C-1′), 116.38 (C-2′), 157.76 (C-3′), 146.42 (C-4′), 115.19 (C-5′), 120.26 (C-6′).

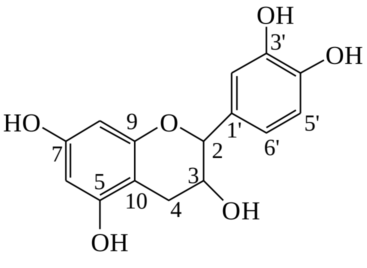

catechin

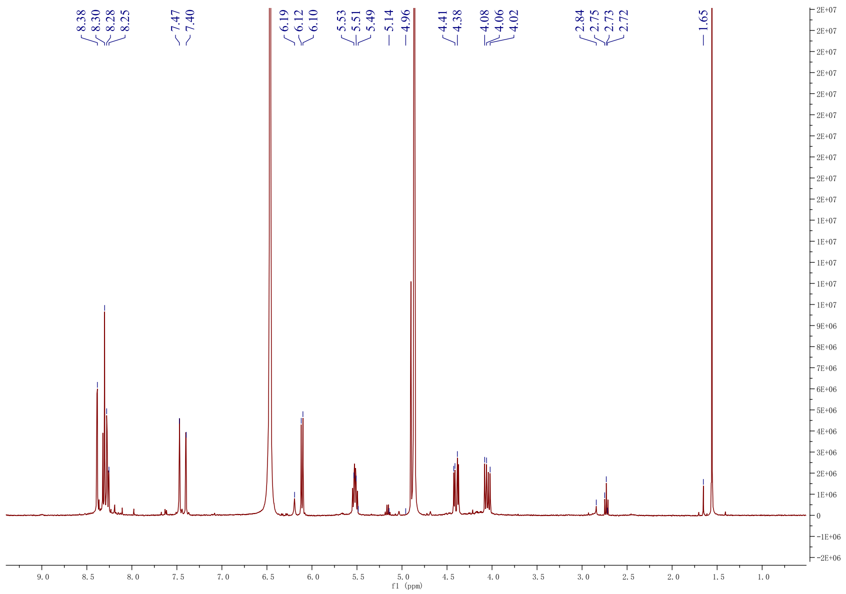

The ^{1}H-NMR spectrum of compound 4

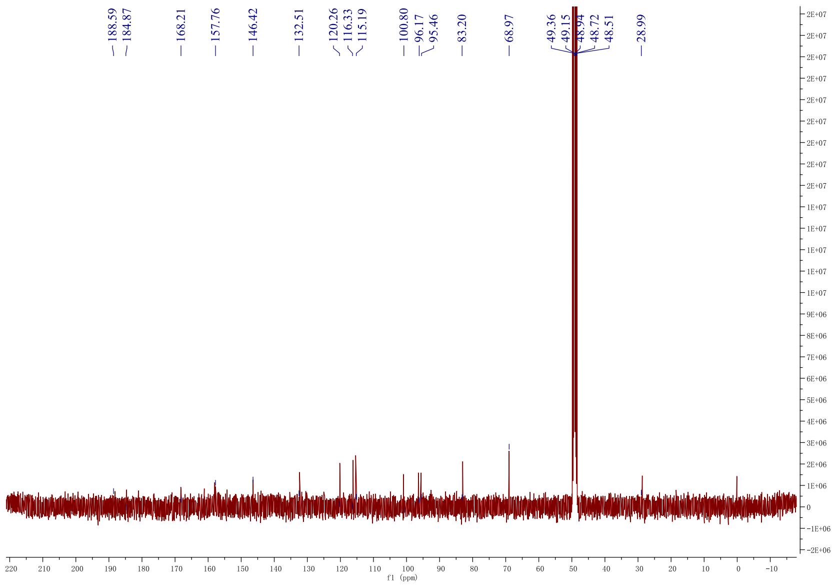

The ^{13}C-NMR spectrum of compound 4

Luteolin (5): ^{1}H-NMR (400 MHz, DMSO-d_6), δ 12.98 (1H, s, 5-OH), 7.43 (1H, br d, 6′-H), 7.41 (1H, br s, 2′-H), 6.90 (1H, d, *J*= 9.0 Hz, 5′-H), 6.46 (1H, d, *J*= 1.8 Hz, 8-H), 6.20 (1H, d, *J*= 1.8 Hz, 6-H), 6.67 (1H, s, 3-H). ^{13}C-NMR (400 MHz, DMSO-d_6): δ 150.15 (C-2), 103.33 (C-3), 182.12 (C-4), 164.35 (C-5), 99.28 (C-6), 164.57 (C-7), 94.29 (C-8), 161.93 (C-9), 104.16 (C-10), 121.96 (C-1′), 116.47 (C-2′), 157.74 (C-3′), 146.19 (C-4′), 113.82 (C-5′), 119.45 (C-6′).

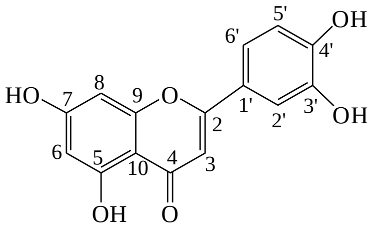

luteolin

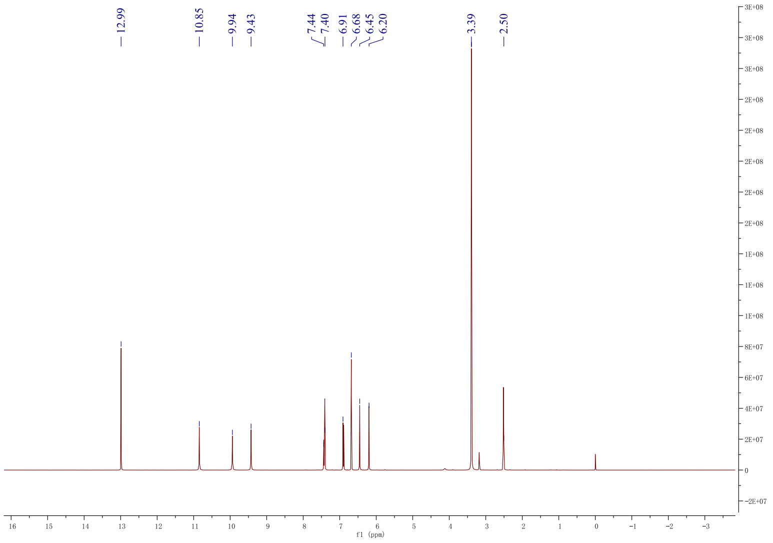

The ¹H-NMR spectrum of compound 5

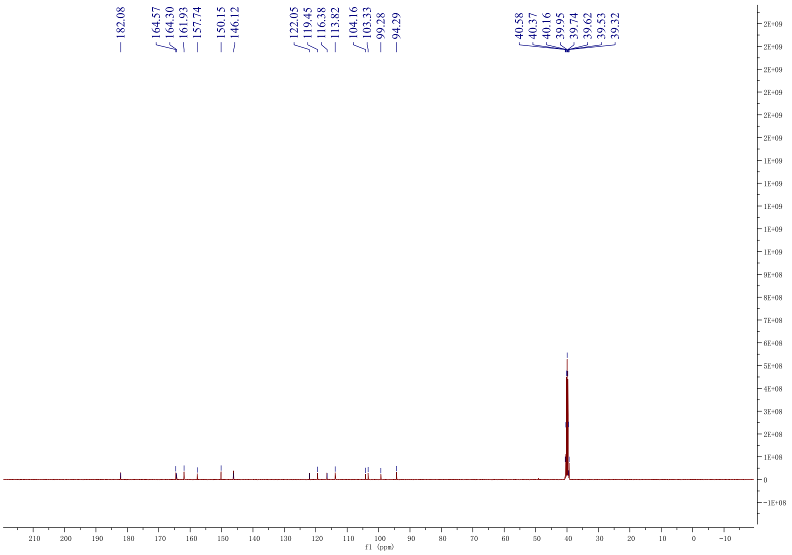

The ¹³C-NMR spectrum of compound 5

Kaempferol (6): ^{1}H-NMR (400 MHz, DMSO-d_6): δ 12.49 (1H, s, 5-OH), 10.80 (1H, s, 7-OH), 10.13 (1H, s, 4-OH), 9.43 (1H, s, 3-OH), 8.05 (2H, d, *J*= 8.8Hz, H-2, 6), 6.93 (2H, d, *J*= 8.8 Hz, H-3′, 5′), 6.44 (1H, d, *J*= 1.5 Hz, H-8), 6.19 (1H, d, *J*= 1.5 Hz, H-6). ^{13}C-NMR (400 MHz, DMSO-d_6): δ 147.27 (C-2), 136.12 (C-3), 176.37 (C-4), 161.18 (C-5), 98.66 (C-6), 164.35 (C-7), 93.94 (C-8), 156.64 (C-9), 103.51 (C-10), 122.13 (C-1′), 129.97 (C-2′&6′), 115.90 (C-3′&5′), 159.65 (C-4′).

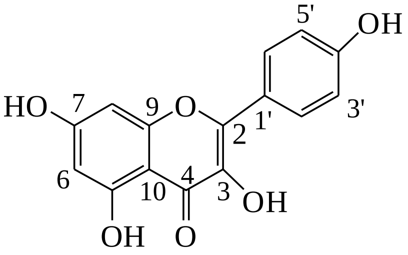

kaempferol

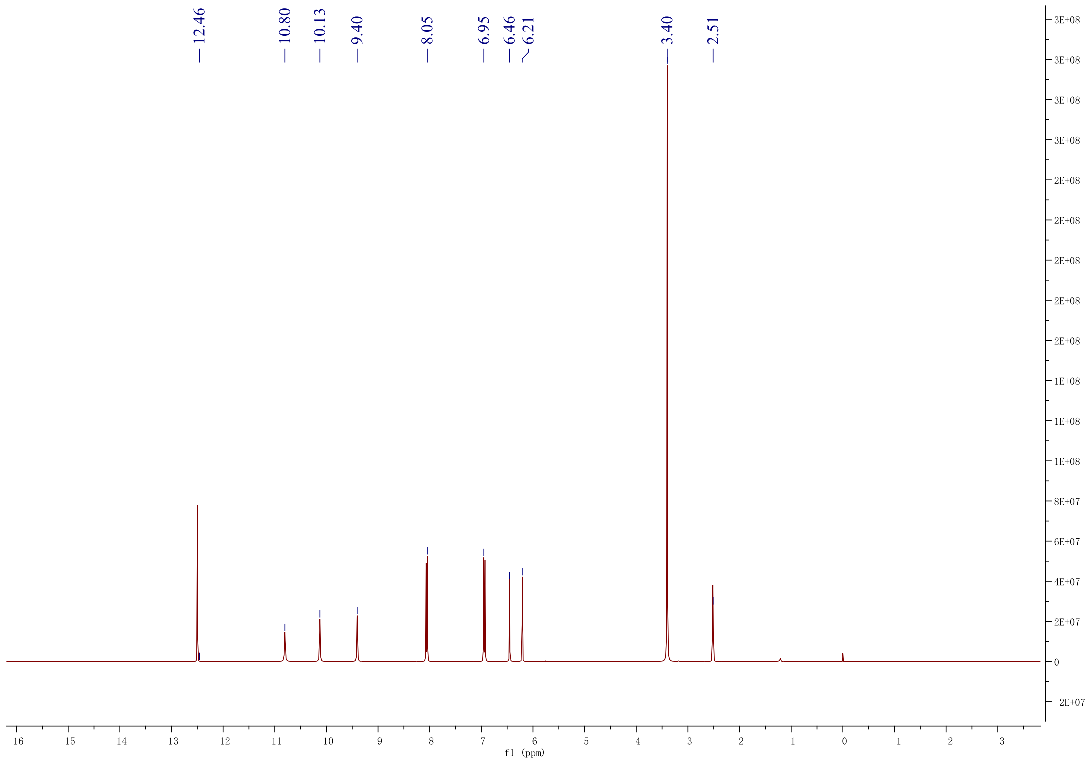

The ^{1}H-NMR spectrum of compound 6

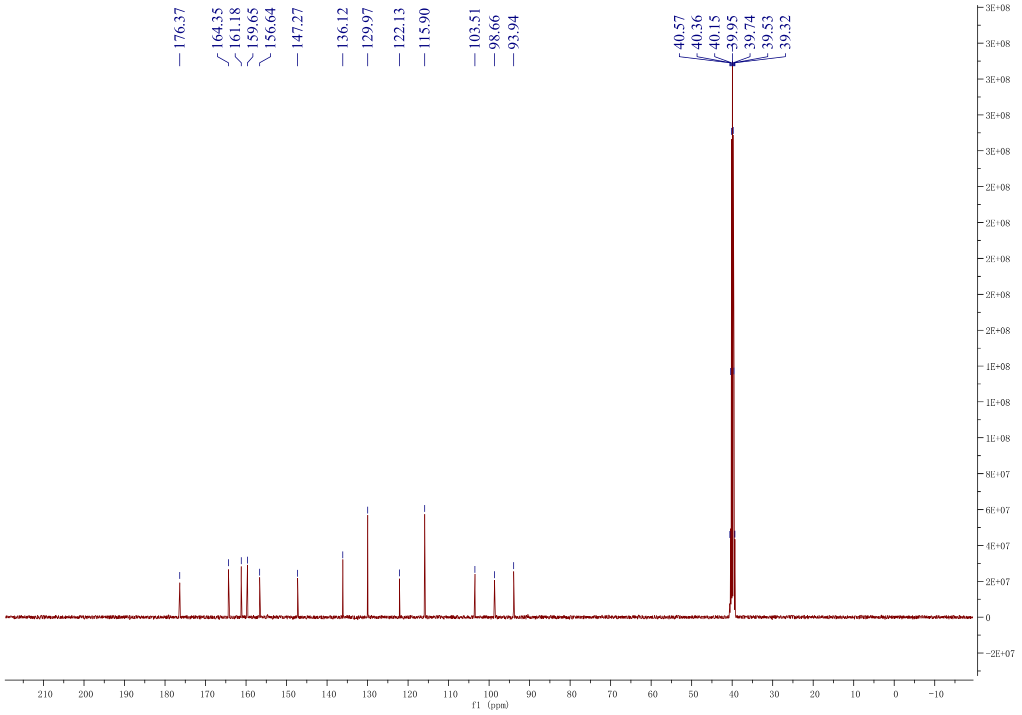

The ^{13}C -NMR spectrum of compound 6

Figure S3. Structural identification of 6 main phenolic compounds in FBPE.

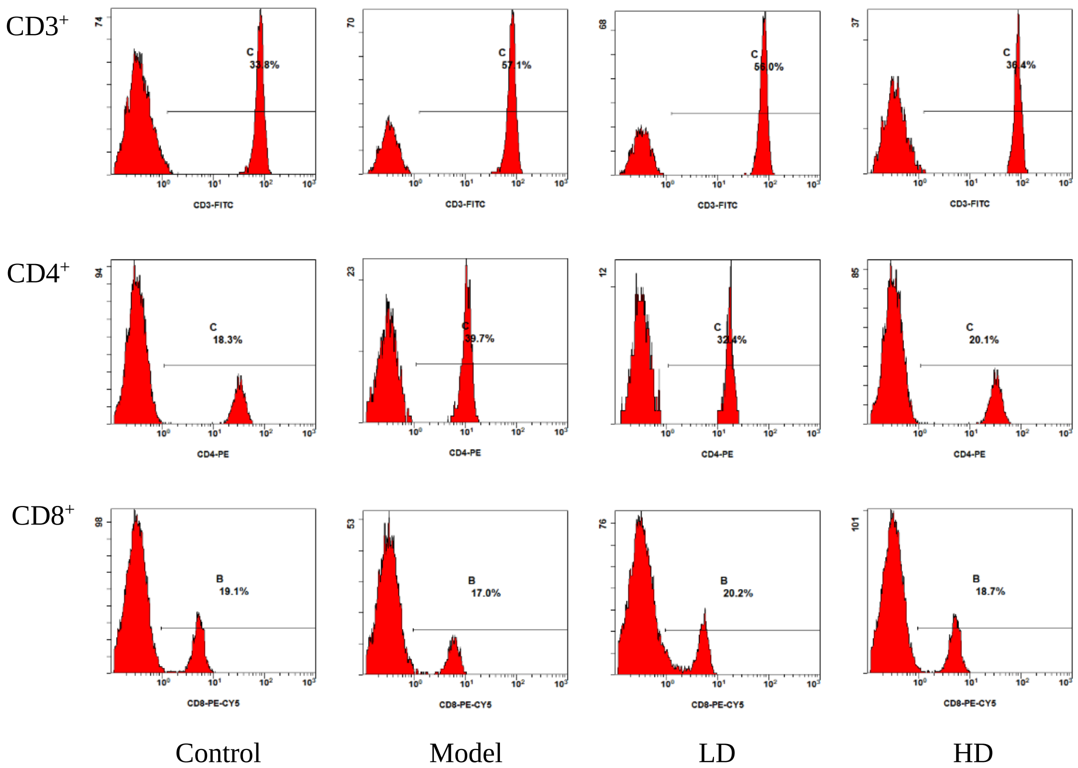

Figure S4. Representative images showing flow cytometry of T cell subsets in different groups of splenocytes.

Table S1 Phenolic compounds of FBPE

**TPC mg gallic acid/g**	**TFC mg rutin/g**	**No.**	**Compounds**	***M*r**	**Ion mode**	**Exptl (m/z)**	**Formula**	**t_R (min)**	**Content (mg/g)**
18.59±1.03	16.35±1.95	1	Chlorogenic acid	354	-	353	$C_{16}H_{18}O_9$	29.53	1.45±0.10
		2	Caffeic acid	180	-	179	$C_9H_8O_4$	30.47	3.47±0.13
		3	Resveratrol	228	-	227	$C_{14}H_{12}O_3$	32.26	5.25±0.51
		4	catechin	290	+	291	$C_{15}H_{14}O_6$	44.85	2.40±0.18
		5	Luteolin	286	-	285	$C_{15}H_{10}O_6$	47.89	9.46±0.12
		7	Kaempferol	286	-	285	$C_{15}H_{10}O_6$	49.10	3.67±0.04

Results presented in the table are expressed as mean±standard deviation (SD) for 3 replications.
